# 924. Pediatric Burns for Low & Middle-Income Countries: Differing Patterns of Epidemiology and Care Seeking Behavior

**DOI:** 10.1093/jbcr/irag033.464

**Published:** 2026-04-06

**Authors:** Dattesh R Dave

**Affiliations:** Shriners Children's Northern California, Sacramento, CA

## Abstract

**Introduction:**

Low-and middle-income (LMIC) countries account for 90% of all reported burns, nevertheless there is a paucity of providers to treat burns. Current studies on burns in LMICs have not evaluated the gap between care seeking and receiving. This gap may be exacerbated by age with pediatric patients suffering more involved areas. This study explores this gap across socioeconomically similar populations in a multi-country population based assessment to inform burn care strategies.

**Methods:**

The Surgeons Over Seas Assessment of Surgical Need (SOSAS) instrument is a cross sectional national, cluster random sampling survey administered in Nepal, Rwanda, Sierra Leone, and Uganda from 2011 to 2014. The survey identifies burn etiology, demographics, timing, disability, and barriers to receiving care.

**Results:**

Among 13 763 individuals surveyed, 896 burns were identified, of which 267 were under the age of 15 (pediatric). Burns of the head and face were more common in the pediatric cohort relative to the adult cohort. Overall, there was an even distribution between flame and scald mechanism in the pediatric cohort while the adult cohort had a significantly higher proportion of flame (73.3% vs 50.2%). The adult cohort more frequently reported their burn to have occurred in the past while the pediatric cohort more frequently reported their burn to be a current condition (28.5% vs 21.78%). Amongst the pediatric cohort disability was more frequently reported in Uganda. Amongst the pediatric cohort lack of money, healthcare providers, and rural living were more commonly reported in Nepal. Rwanda had the highest proportion of individuals seeking and receiving care (91.6% vs 88.5%). Seeking care and having care met was only associated with pediatric versus adult cohorts in Rwanda. Logistic regression analysis demonstrated that the adult cohort were more likely to seek care (OR 0.1 – 1.4) and more likely to have their care met in Sierra Leone (OR 0.1 – 1.2).

**Conclusions:**

There was significant variability in burn mechanism, anatomic location, timing, and disability between pediatric and adult cohorts across the four countries. Care seeking and care receiving was most associated with the adult cohort than the pediatric cohort.

**Applicability of Research to Practice:**

Many burn surgeons participate in "mission" trips. This model has come into question as capacity building has been shown to empower local surgeons and build towards longitudinal burn care. Understanding baseline prevalence, socioeconomic, and burn type distribution at the pediatric level can help better match burn need and burn care.

**Funding for the study:**

N/A.

